# Supplementary material for: Fast kinetics of multivalent intercalation chemistry enabled by solvated magnesium-ions into self-established metallic layered materials
Source: Nat Commun. 2018 Nov 30;9:5115. doi: 10.1038/s41467-018-07484-4 (PMC6269537; doi:10.1038/s41467-018-07484-4)
Supplement: Supplementary file 1 — Supplementary Information [file 41467_2018_7484_MOESM1_ESM.pdf]

Supporting Information

Fast kinetics of multivalent intercalation chemistry enabled by solvated magnesium-ions into self-established metallic layered materials

Zhenyou Li et al

Supplementary Figures

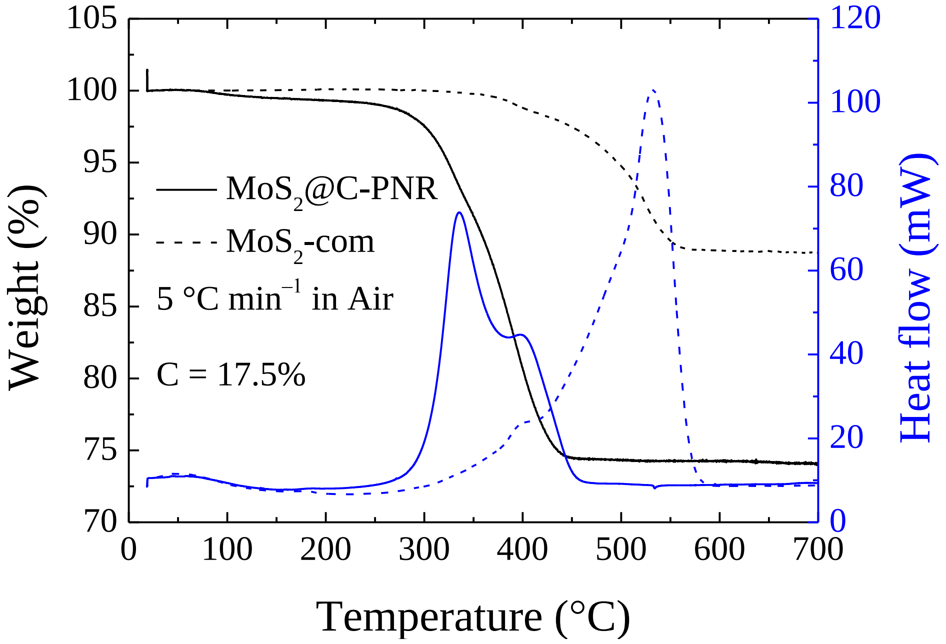

Supplementary Fig. 1 TGA including the heat flow of MoS_2@C-PNR and MoS_2-com.

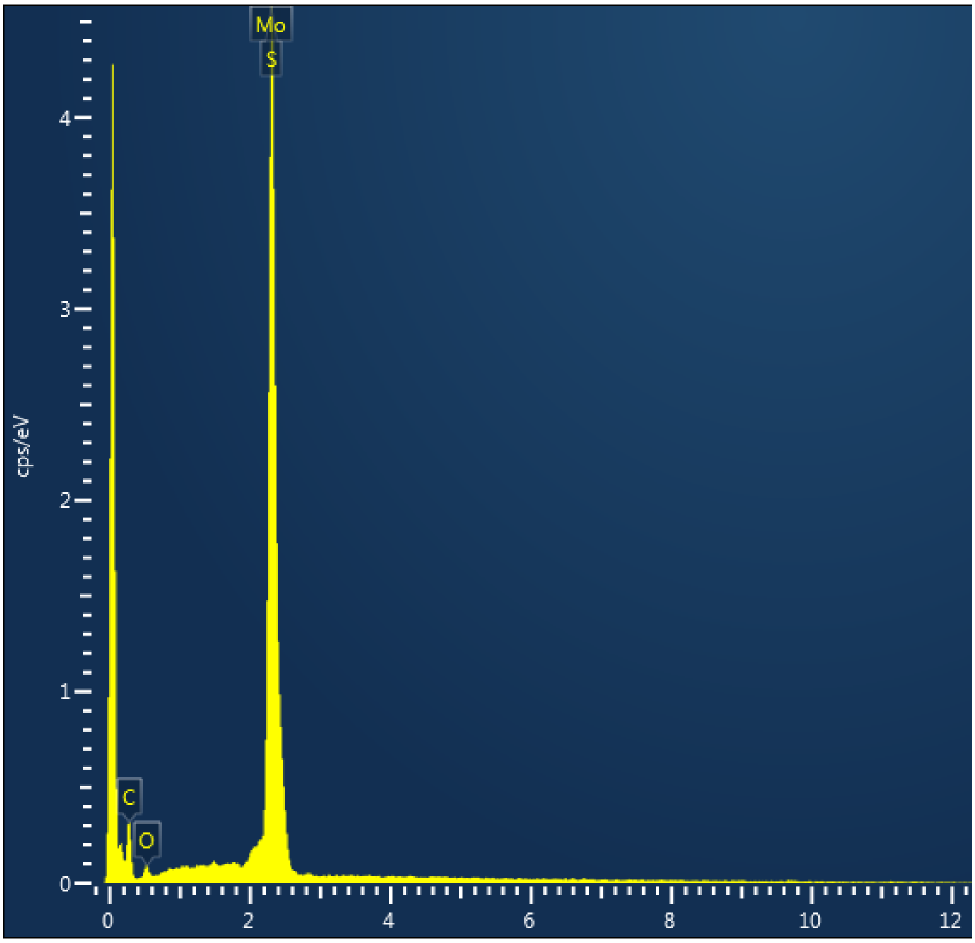

Supplementary Fig. 2 EDX spectrum of MoS_2@C-PNR in the square area shown in Fig. 1d.

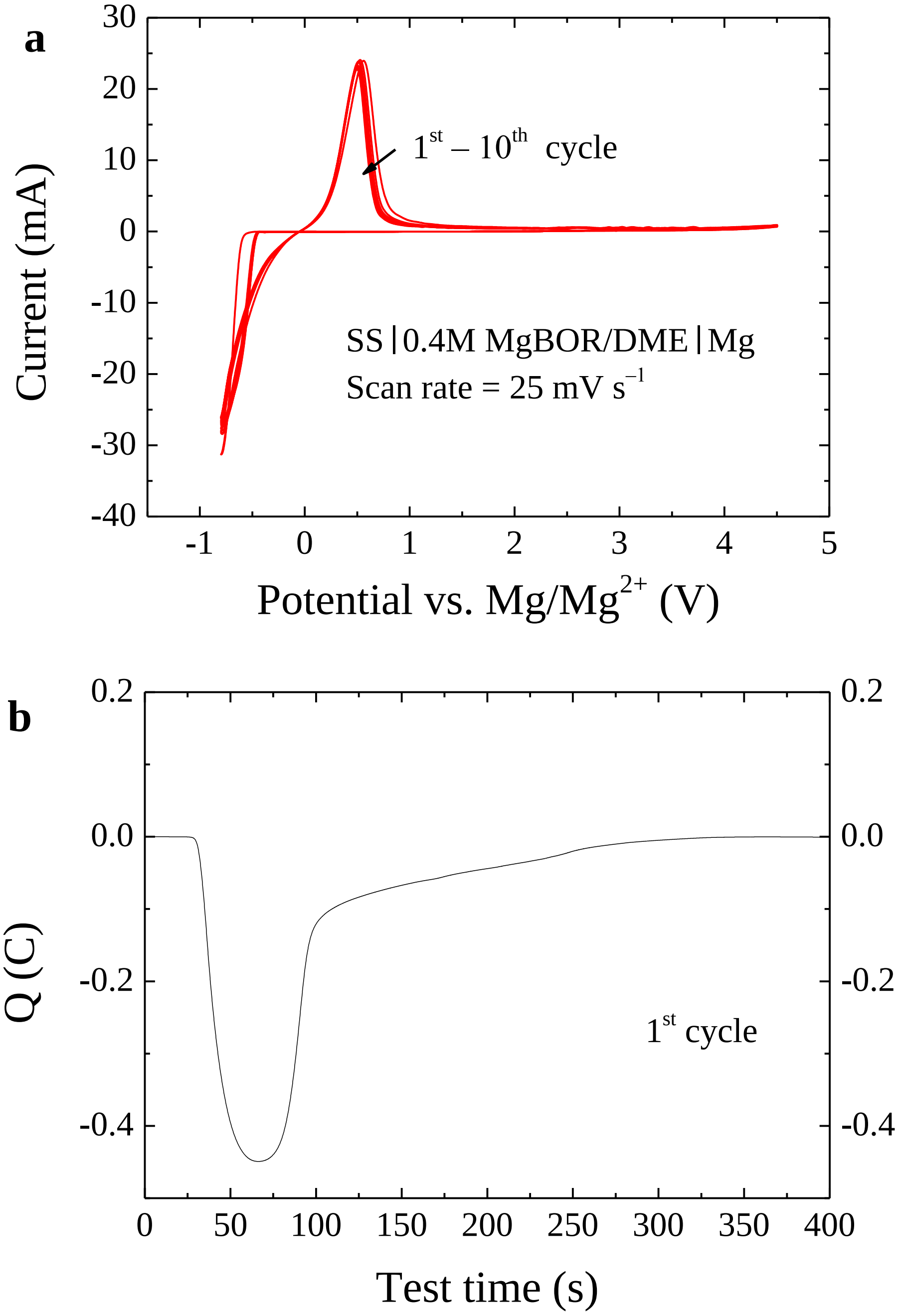

Supplementary Fig. 3 Plating and stripping of Mg from MgBRO electrolyte. **a** CV at 25 mV s^{-1} using stainless steel (SS) as the working electrode, 0.4 M MgBOR in DME as electrolyte and Mg as the counter electrode. **b** The electric charge accumulation with time for the first cycle. There is tiny amount of net charge after a complete plating/stripping process, indicating the high reversibility of the electrolyte.

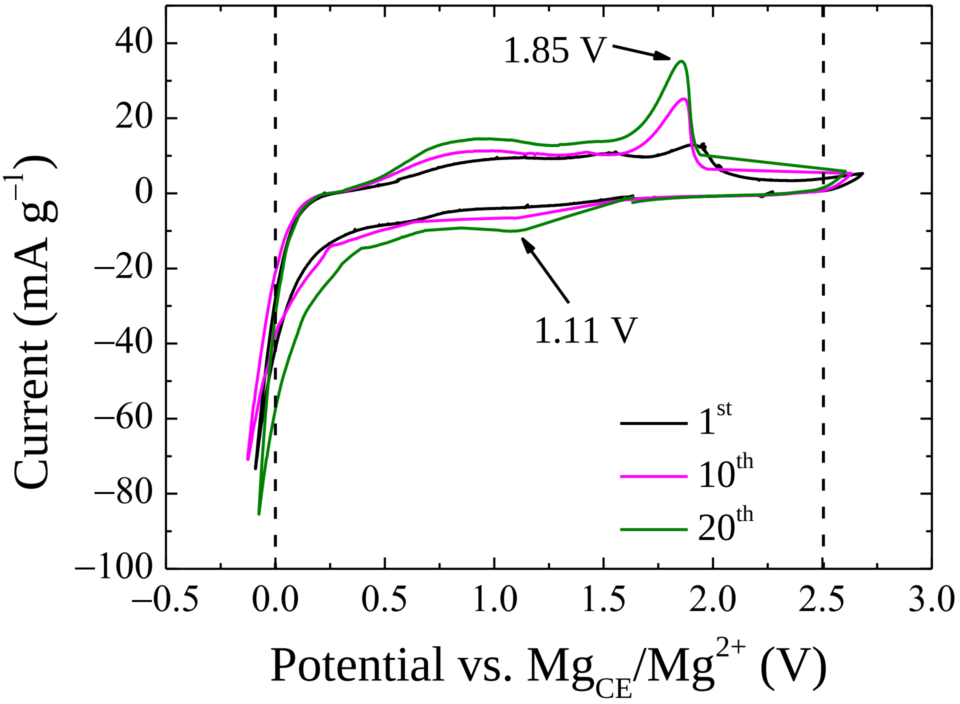

Supplementary Fig. 4 Three-electrode CV of the MoS_2@C-PNR electrode: Potential vs. Mg_{CE}.

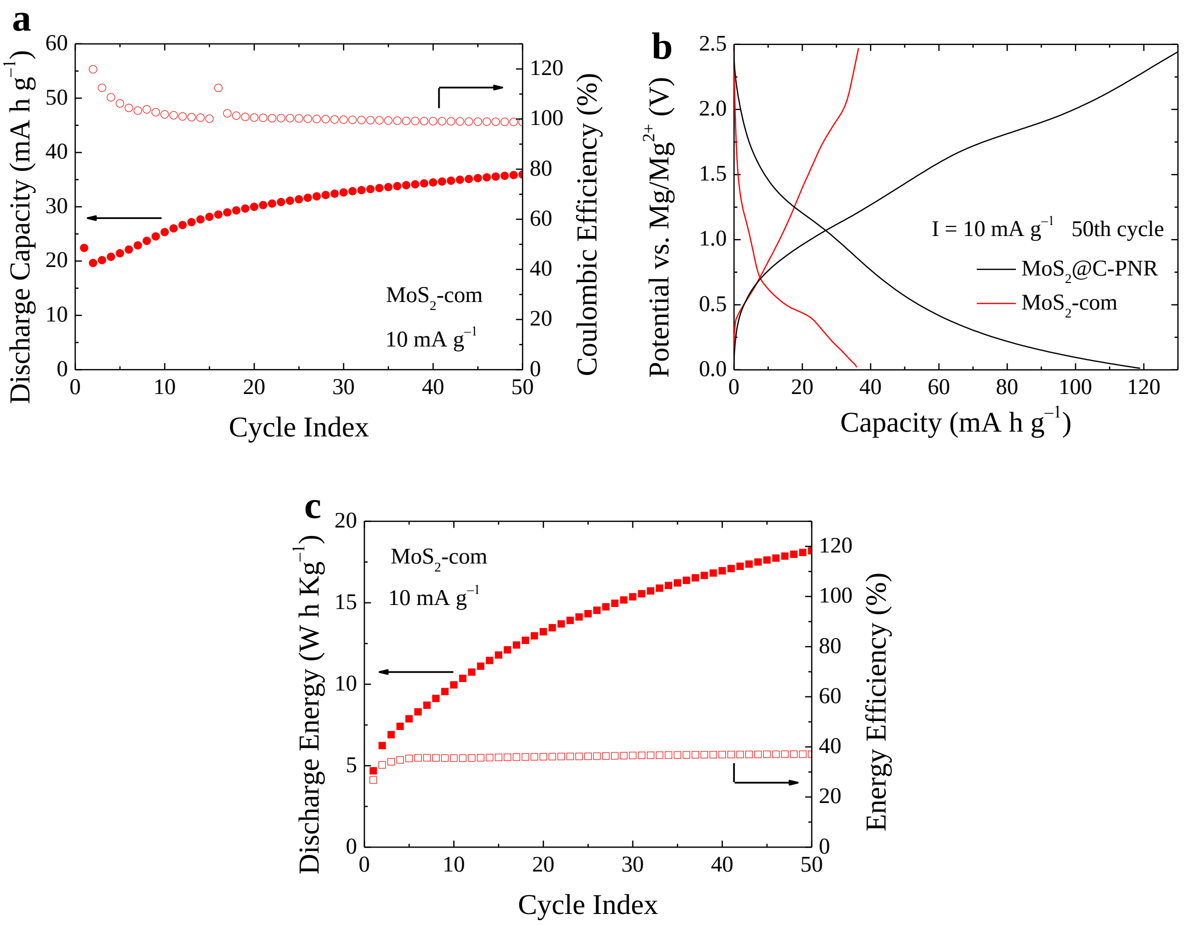

Supplementary Fig. 5 Electrochemical performance of MoS_2-com electrode. **a** Cycling stability of MoS_2-com at 10 mA g^{-1}. **b** Charge-discharge profile comparison of MoS_2@C-PNR and MoS_2-com at 50th cycle. **c** Specific energy and energy efficiency of MoS_2-com at 10 mA g^{-1}.

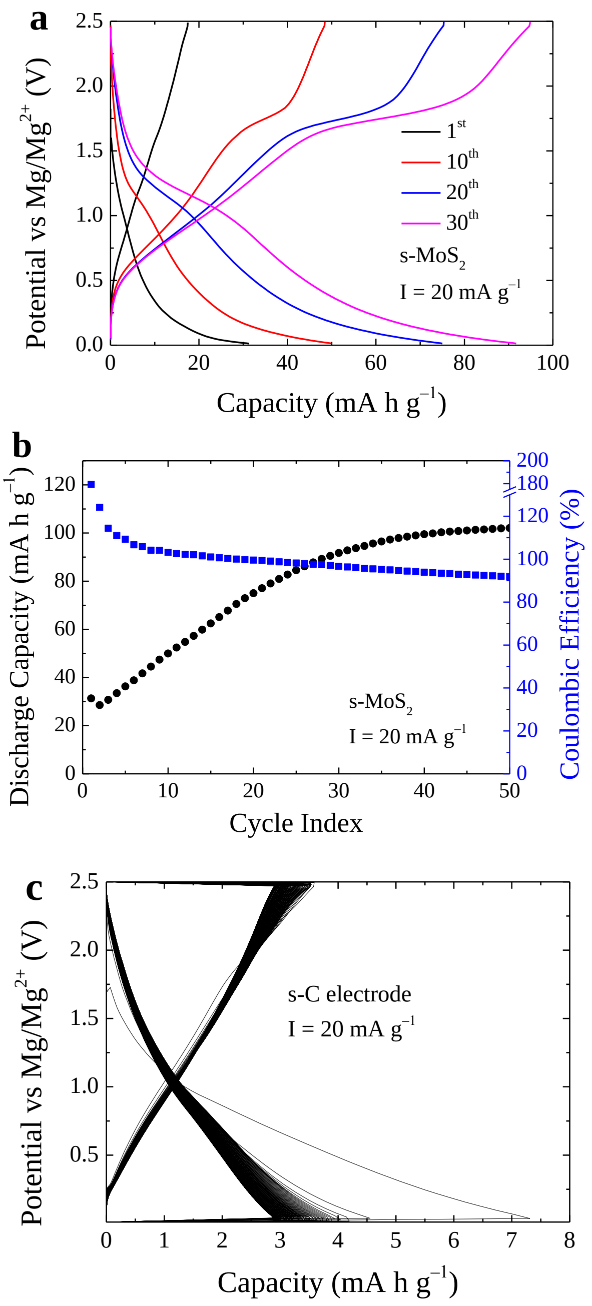

Supplementary Fig. 6 Battery performance of s-MoS_2 and s-C electrodes. **a** Charge-discharge profile and **b** cycling stability of s-MoS_2 electrode at 20 mA g^{-1}. **c** Charge-discharge profile of s-C electrode at 20 mA g^{-1}. Detailed discussion in Supplementary Note 1.

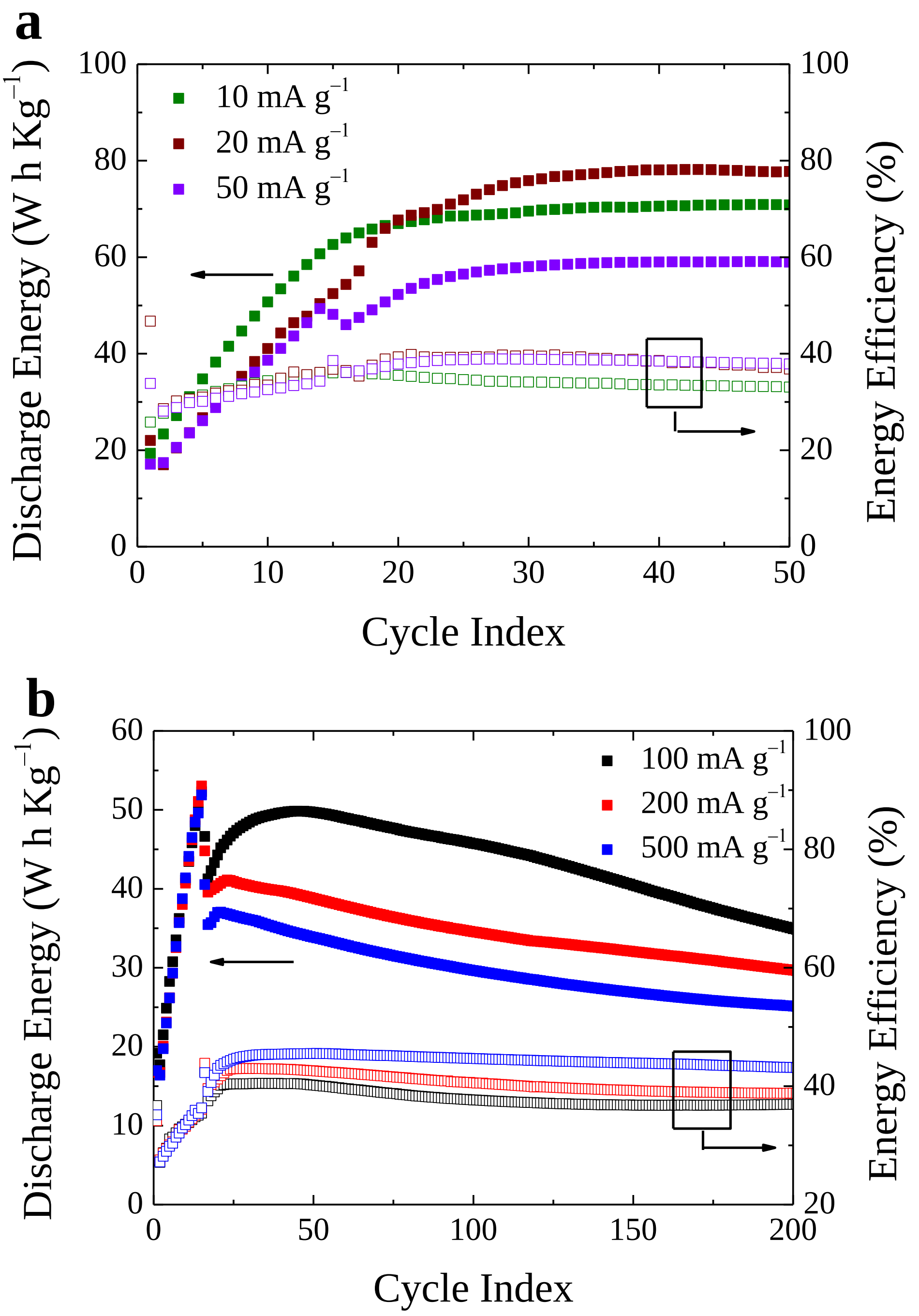

Supplementary Fig. 7 Specific energy and energy efficiency of the MoS_2@C-PNR electrode at various current rates. **a** Specific energy; **b** energy efficiency.

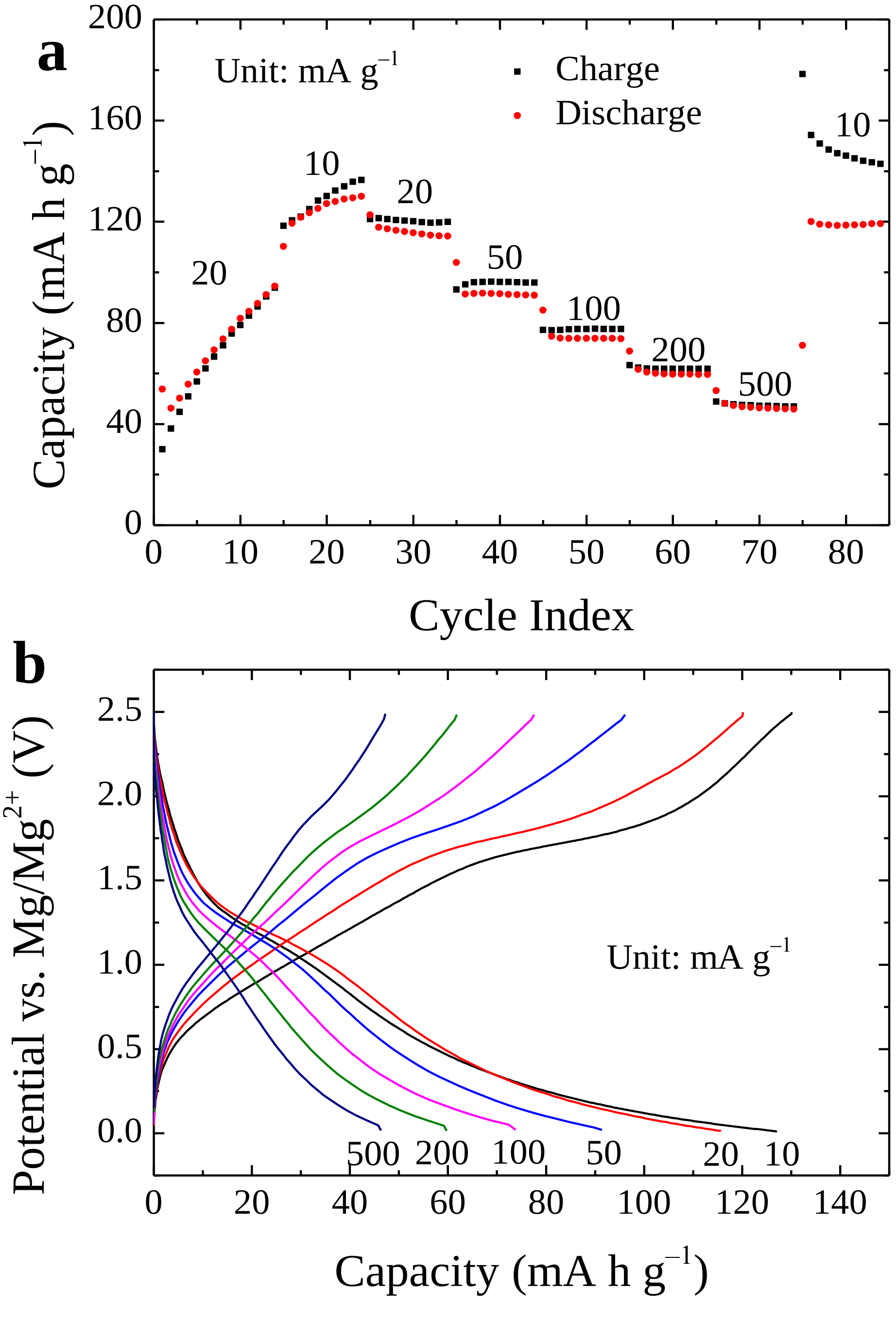

Supplementary Fig. 8 Rate capability of MoS_2@C-PNR. **a** Rate performance; **b** the corresponding charge-discharge profiles. The cell is also activated at 20 mA g^{-1} for 15 cycles before applying different current rate.

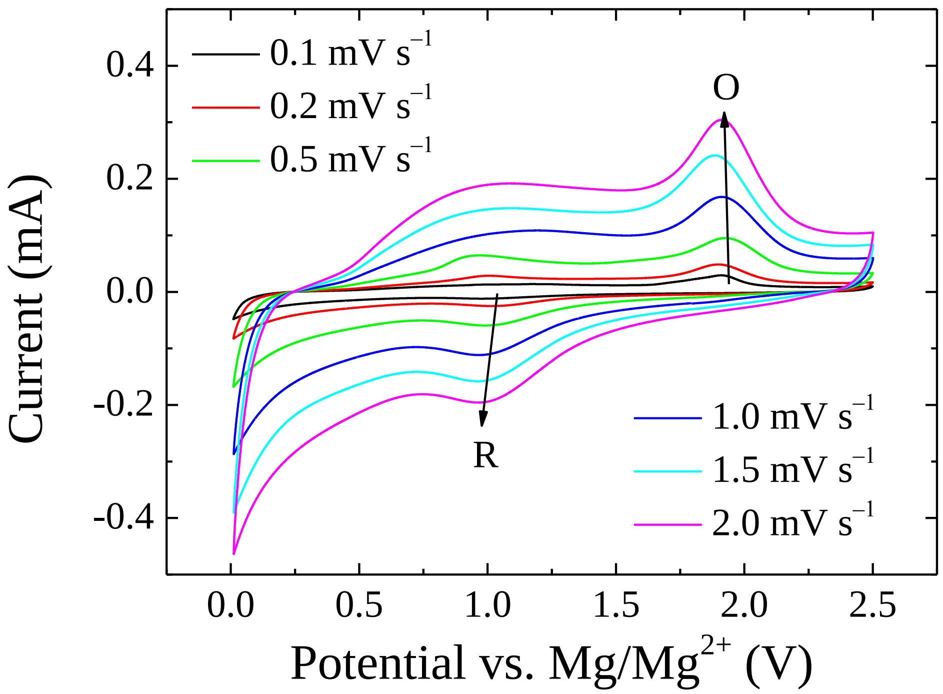

Supplementary Fig. 9 CV of MoS_2@C-PNR electrode at different scan rate. The cell was activated by galvanostatic cycling at 20 mA g^{-1} for 15 cycles before CV test.

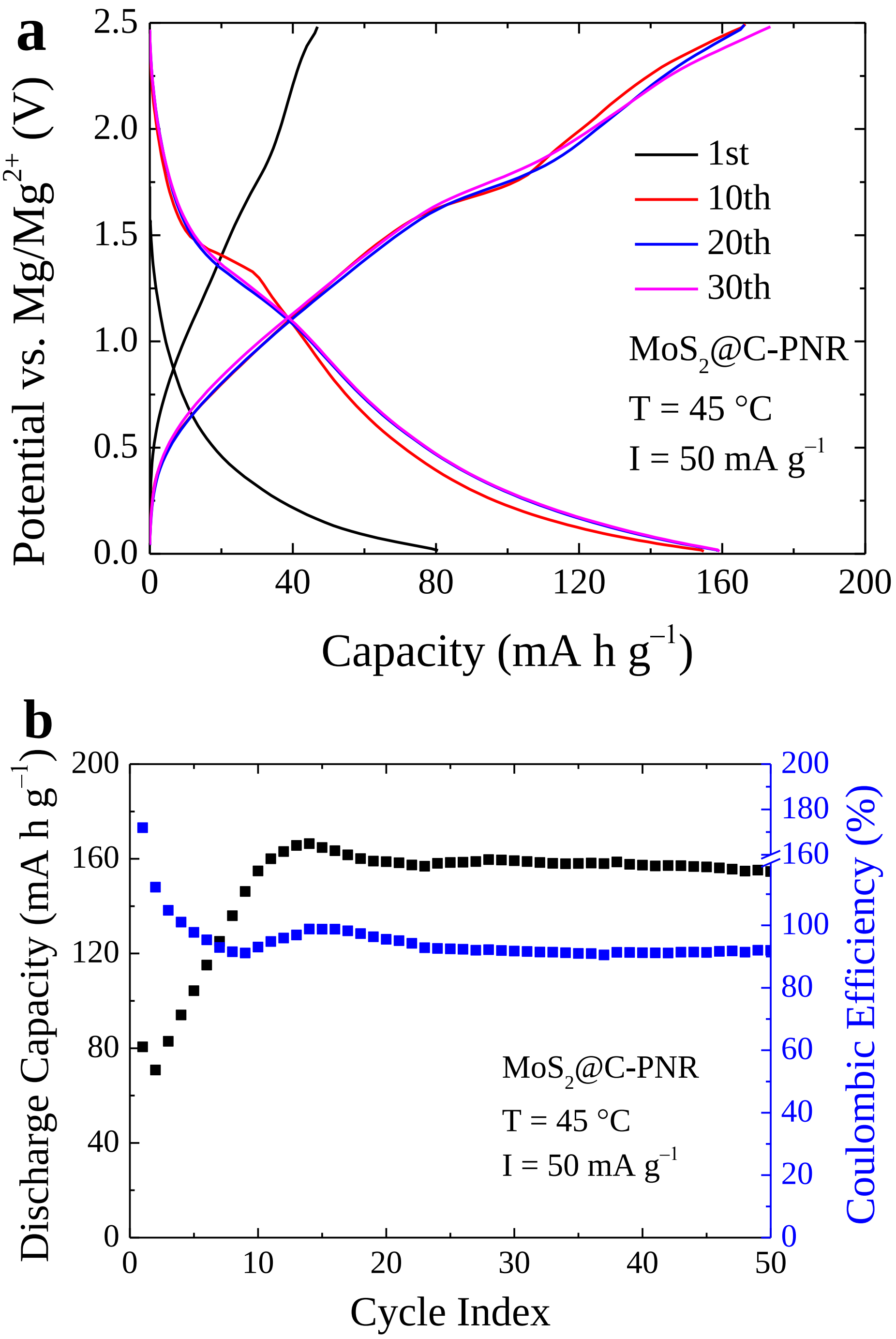

Supplementary Fig. 10 Battery performance of MoS_2@C-PNR electrode at elevated temperature of T = 45 °C. **a** Charge-discharge profile at I = 50 mA g^{-1}; **b** the corresponding cycling stability.

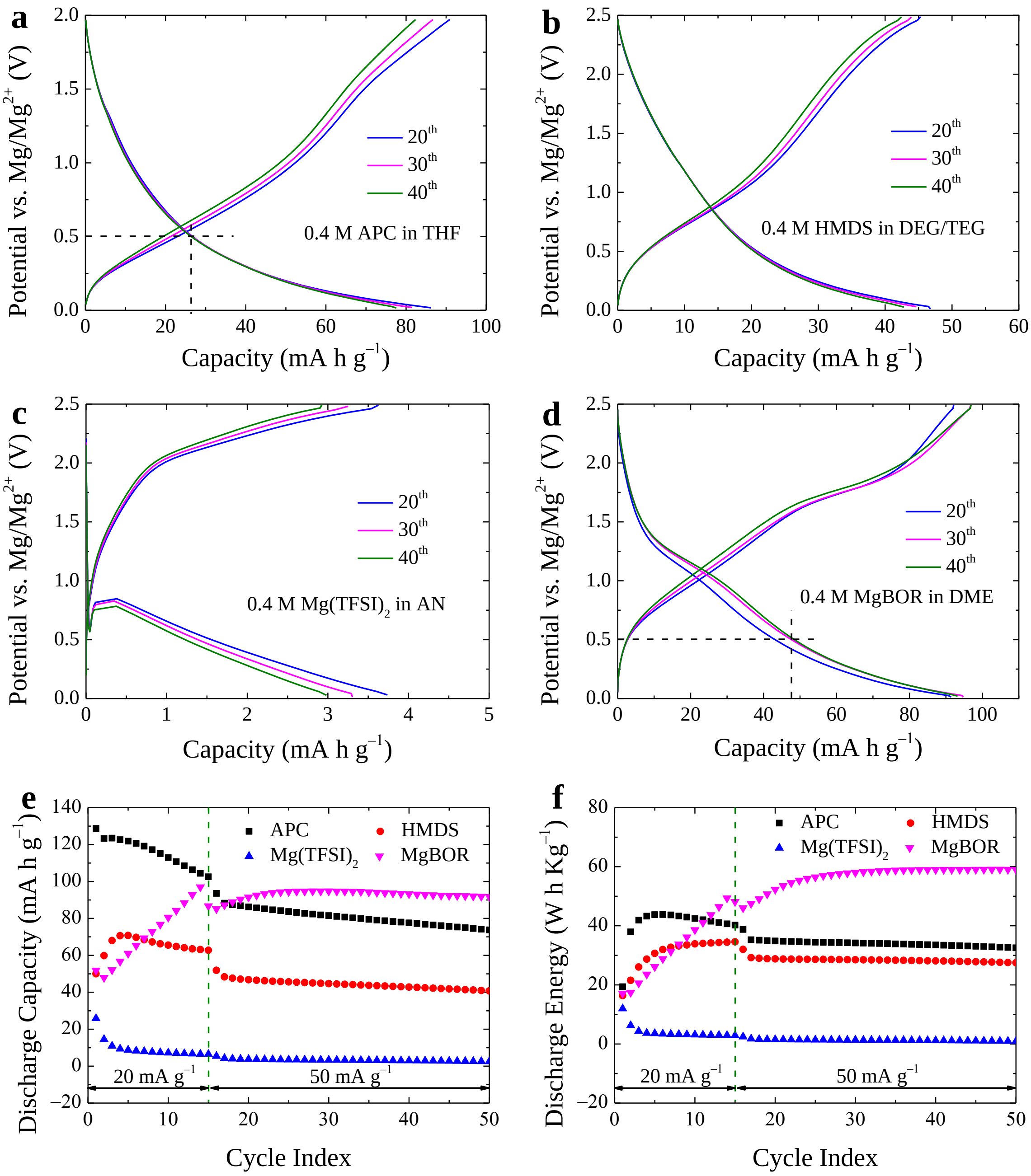

Supplementary Fig. 11 Battery performance of MoS_2@C-PNR electrode with different electrolytes. Charge-discharge profiles of the MoS_2@C-PNR electrode with **a** 0.4 M APC in THF; **b** 0.4M HMDS in DEG/TEG; **c** 0.4 M $Mg(TFSI)_2$ in AN; **d** 0.4 M MgBOR in DME. **e** A comparison of their discharge capacity and **f** discharge energy. All the cells are activated at 20 mA g^{-1} for 15 cycles before applying a high current of 50 mA g^{-1}.

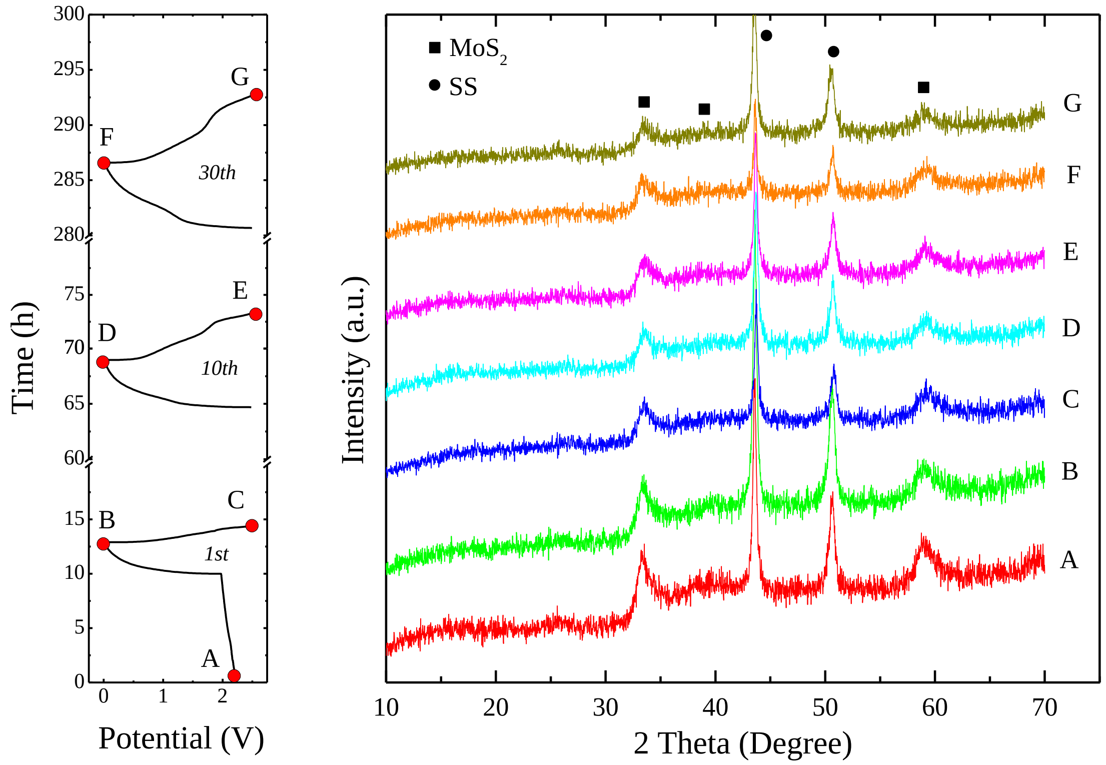

Supplementary Fig. 12 *Ex situ* XRD of MoS_2@C-PNR electrodes at specific charge states indicated in the dis-/charge profile.

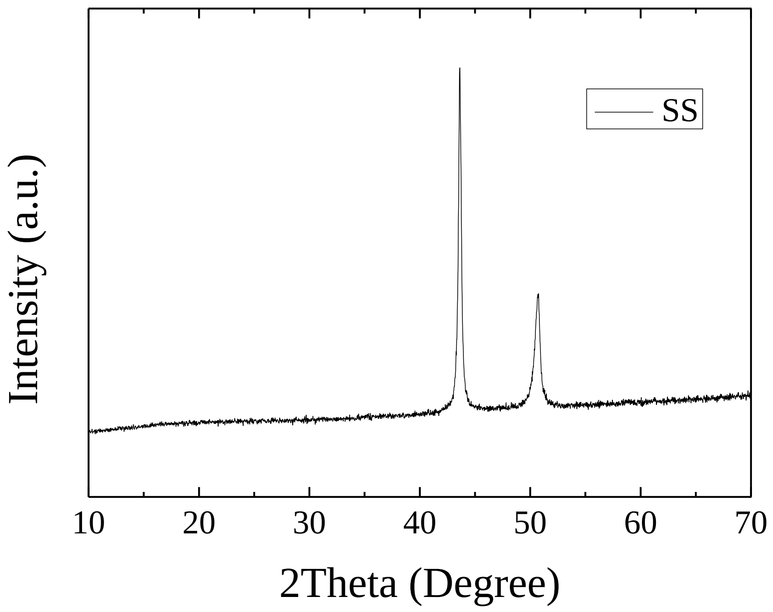

Supplementary Fig. 13 XRD of blank stainless steel (SS) current collector.

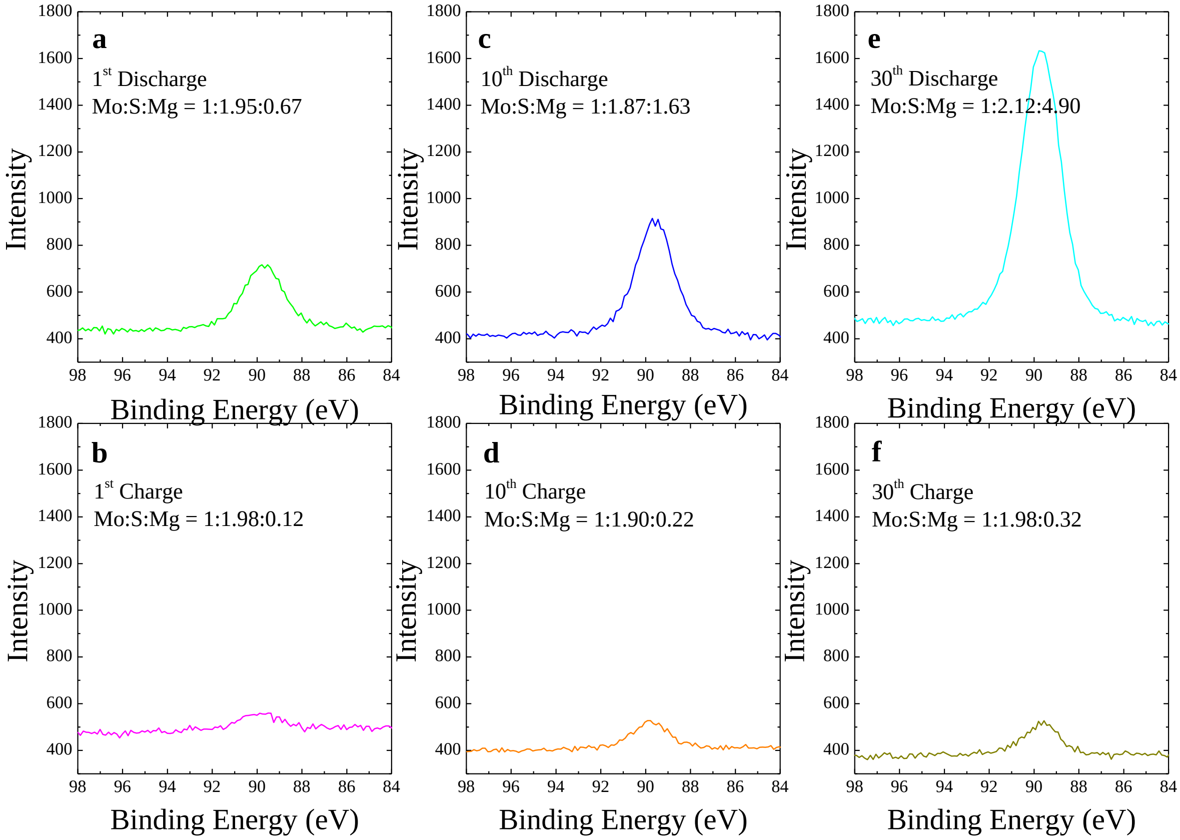

Supplementary Fig. 14 *Ex-situ* XPS Mg2s spectrums of MoS_2@C-PNR electrodes at specific charge states. **a** 1st Discharge; **b** 1st Charge; **c** 10th Discharge; **d** 10th Charge; **e** 30th Discharge; **f** 30th Charge. It should be mentioned here that there is still trace amount of electrolyte remained in the samples according to the B1s spectrums in Supplementary Fig. 15. Considering electrolyte residuals, the Mg contribution from the electrolyte has been excluded based on the B concentration in each sample. Since MoO_3 residues also contribute to the Mo content, the Mo^{6+} contribution was excluded based on the fitting peak area. In addition, the Mg:Mo:S ratios only reflect the element components in the very surface part (~4–6 nm in depth). The large Mg/Mo ratio of the surface could be attributed to the limited Mg^{2+} mobility but much faster electrons transfer of the host. For comparison, the Mo:S:Mg ratio for the pristine sample is also determined which is 1:2.01:0.

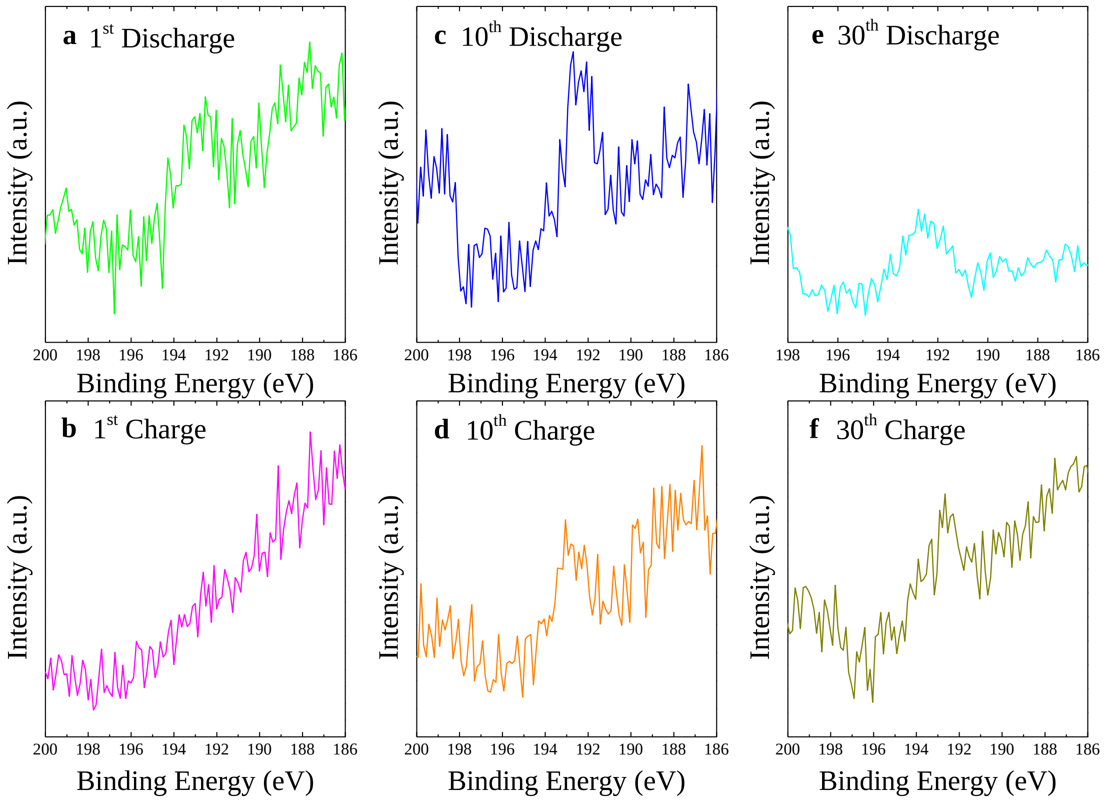

Supplementary Fig. 15 *Ex-situ* XPS B1s spectrums of MoS_2@C-PNR electrodes at specific charge states. **a** 1st Discharge; **b** 1st Charge; **c** 10th Discharge; **d** 10th Charge; **e** 30th Discharge; **f** 30th Charge.

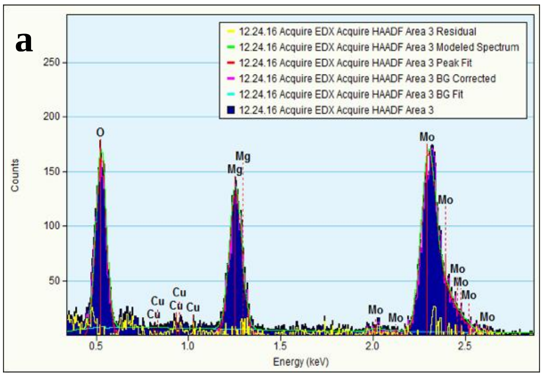

30th discharged (Fig. 4e, area 2)		
**Element**	Weight %	Atomic %
**O(K)**	33.9	58.5
**Mg(K)**	17.6	20.0
**S(K)**	13.1	11.3
**Mo(K)**	35.5	10.2

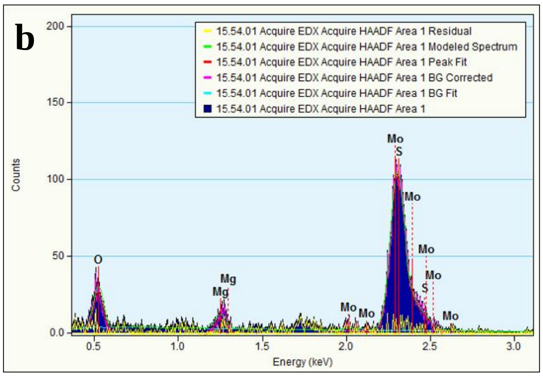

30th charged (Fig. 4f, area 1)		
**Element**	Weight %	Atomic %
**O(K)**	15.2	38.2
**Mg(K)**	3.4	5.6
**S(K)**	26.3	33.0
**Mo(K)**	55.1	23.1

Supplementary Fig. 16 Quantification of Mo:Mg:S:O by EDX spectrum of Mo-K, S-K, O-K, and Mg-K at reacted region. **a** area 2 in Fig. 4c (30th discharged state, reacted region), and **b** area 1 in Fig. 4d (30th charged state). Detailed discussion in Supplementary Note 2.

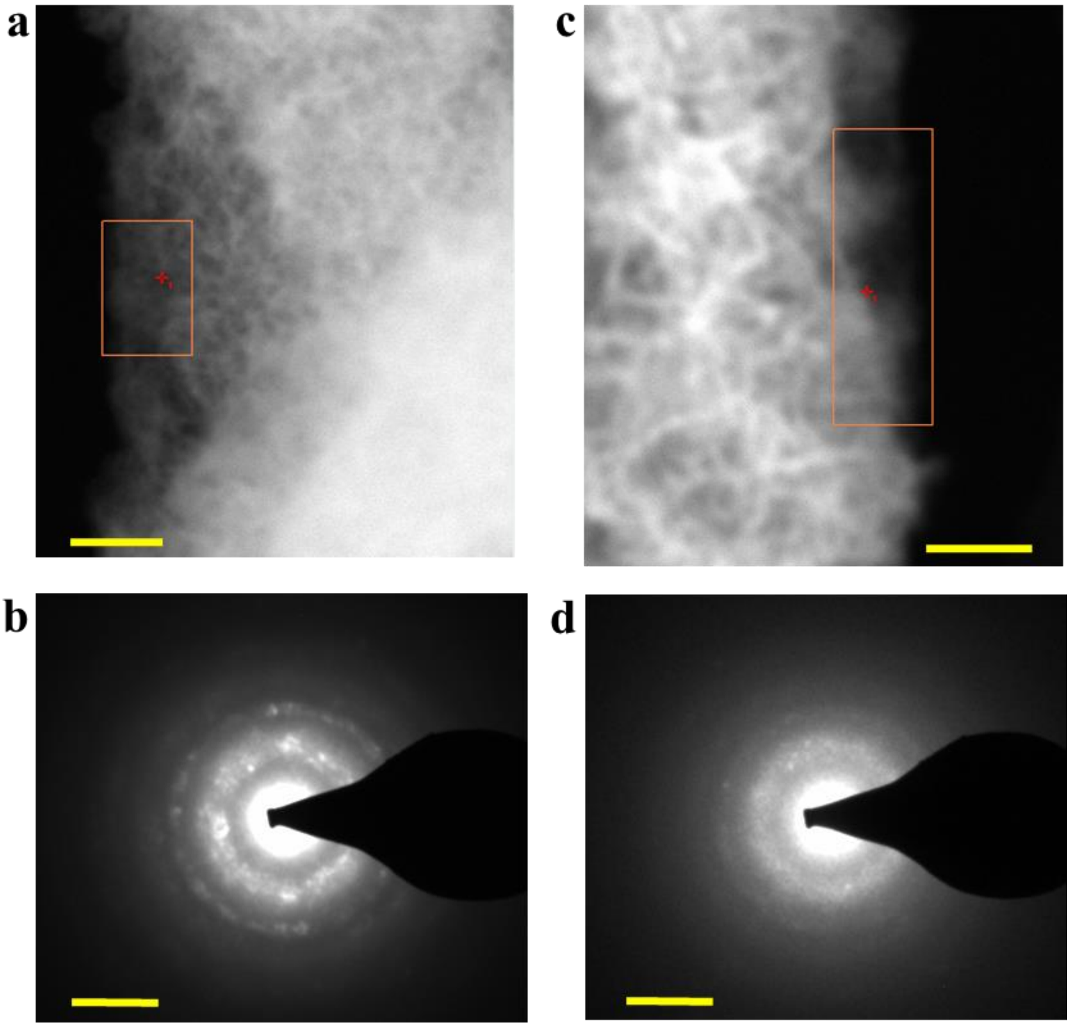

Supplementary Fig. 17 Acquisition area of 4DSTEM (**a**, **c**, orange boxes) for the STEM-RDF results. **a** and **b** are corresponding to 30th charged sample, **c** and **d** are from 30th discharged sample. **b** and **d** are typical diffraction pattern corresponding to the location indicated by the red crosses in **a** and **c** respectively. The scale bars in **a** and **c** correspond to 100 nm, in **c** and **d** to 5 1/nm.

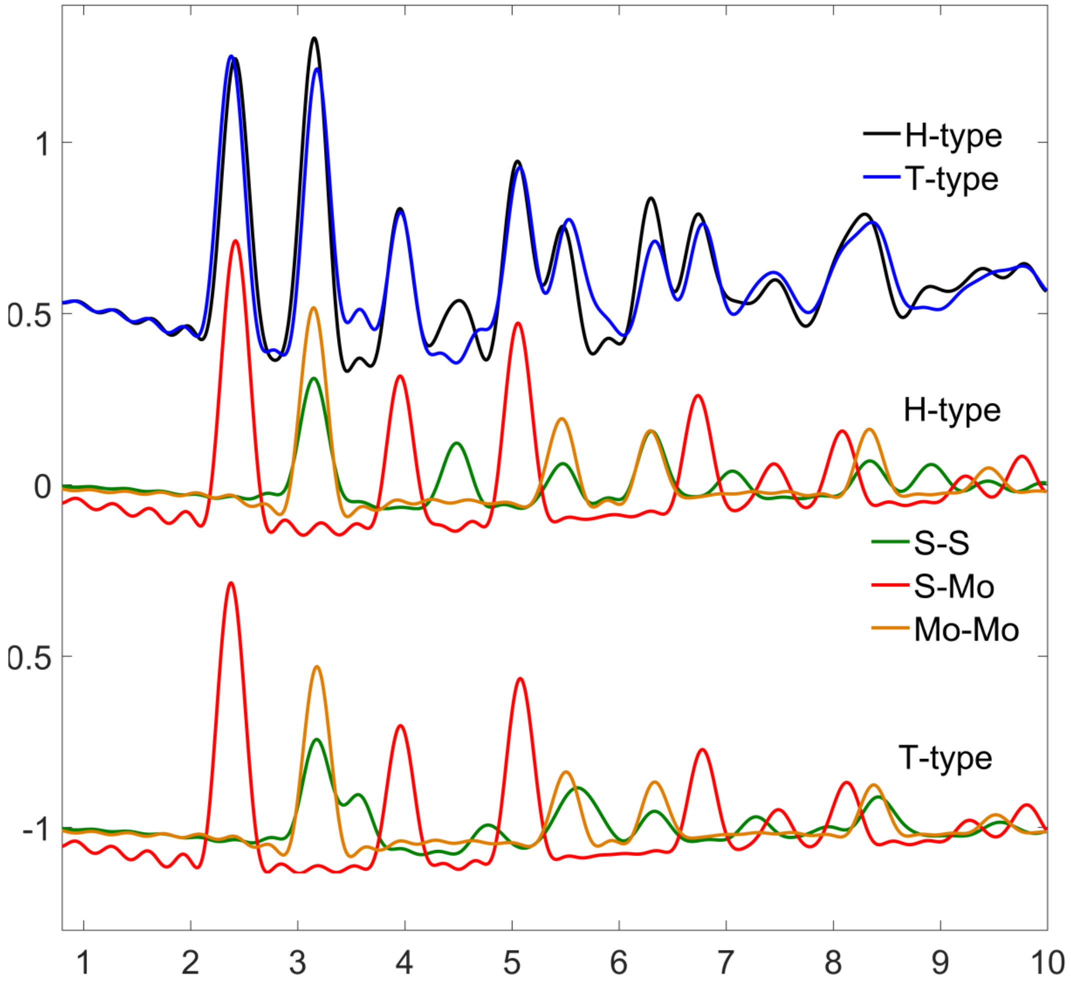

Supplementary Fig. 18 Simulated PDFs of single layer H-/T-type MoS_2 structures. Top: PDF of H- (black line)/T-PDFs (blue line). Middle: partial PDFs of H-type MoS_2, S-S (green), S-Mo (red) and Mo-Mo (orange). Bottom: partial PDFs of T-type MoS_2, following the same colour scheme.

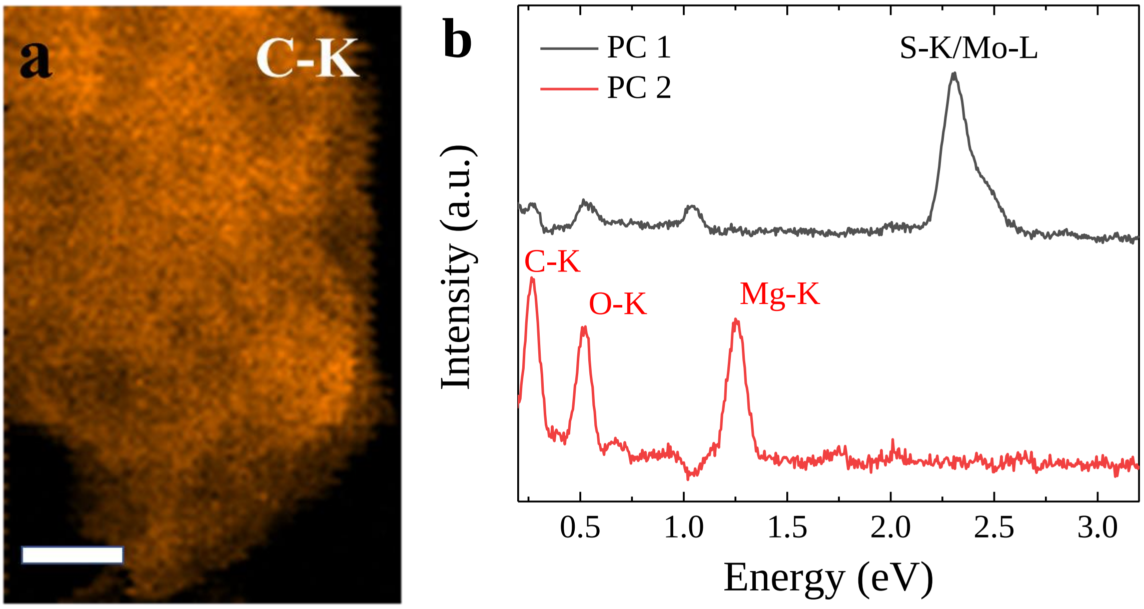

Supplementary Fig. 19 EDX C-K mapping and PCA of MoS_2@C-PNR at 30th discharged state corresponding to Fig.5a. **a** C-K mapping (scale bar = 50 nm); **b** C signal included PCA components of the EDX spectral image. PCA components show the C, O and Mg correlation but anti-correlation to Mo/S.

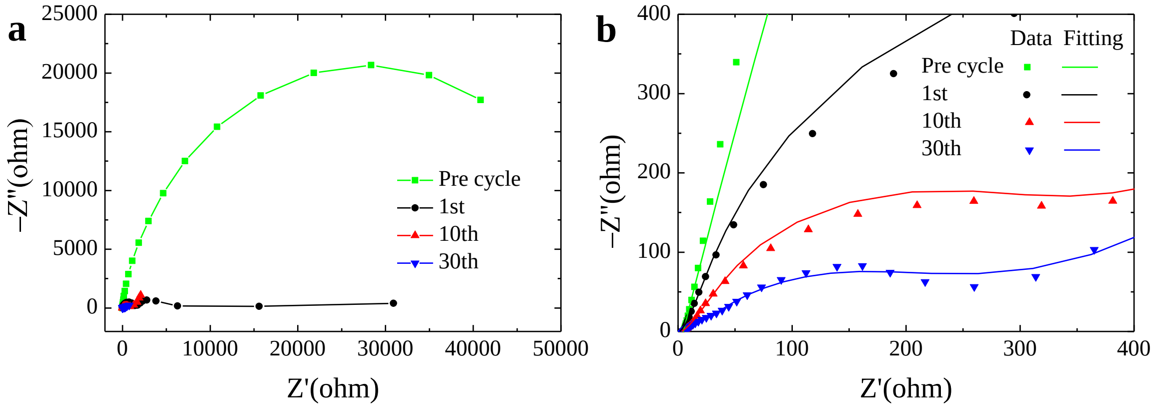

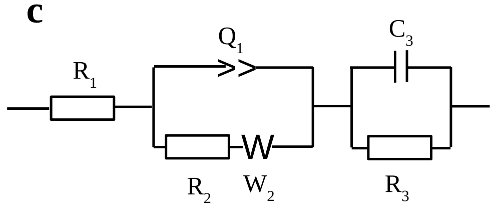

d

State	R1(ohm)	R2(ohm)	R3(ohm)
Pre cycle	–0.8	44167	5.4
1st	1.3	873.6	866.3
10th	11.3	640.5	209.6
30th	15.8	278.4	109.6

Supplementary Fig. 20 EIS of MoS_2@C-PNR at electrodes after specific cycles in the frequency range of 1 MHz to 0.1 Hz. **a** Whole range of the data; **b** data at high to medium frequency range and corresponding fittings; **c** equivalent circuit (detailed discussion in Supplementary Note 3); and **d** calculated parameters in the equivalent circuit. The cell was running at 20 mA g^{-1} for cycling.

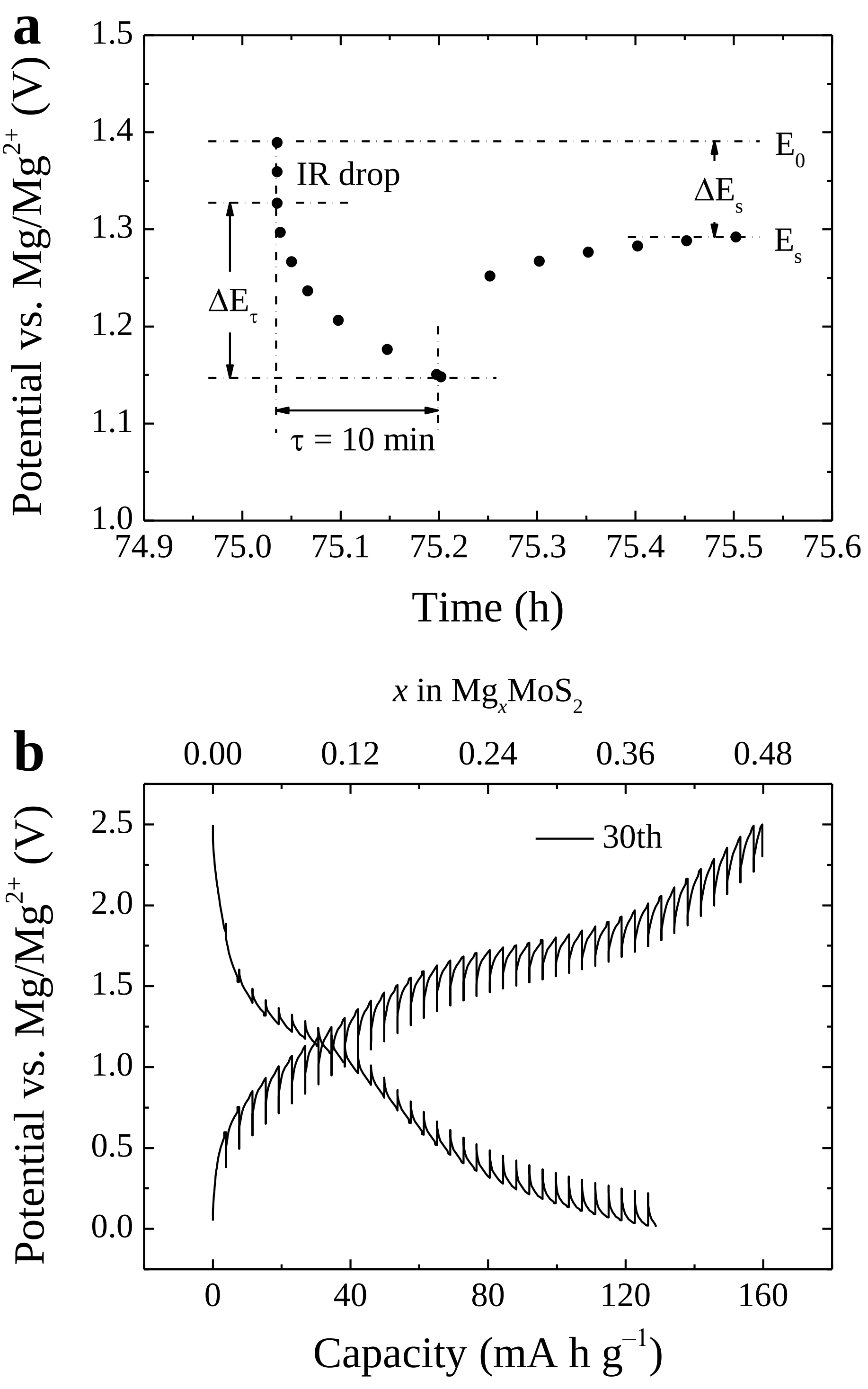

Supplementary Fig. 21 GITT of MoS_2@C-PNR at electrodes at different states. **a** Typical potential response curve, conducted at constant current pulse of 20 mA g^{-1} for 10 min followed by a relaxation period of 20 min; **b** GITT curve at 30th cycle. Detailed discussion in Supplementary Note 4.

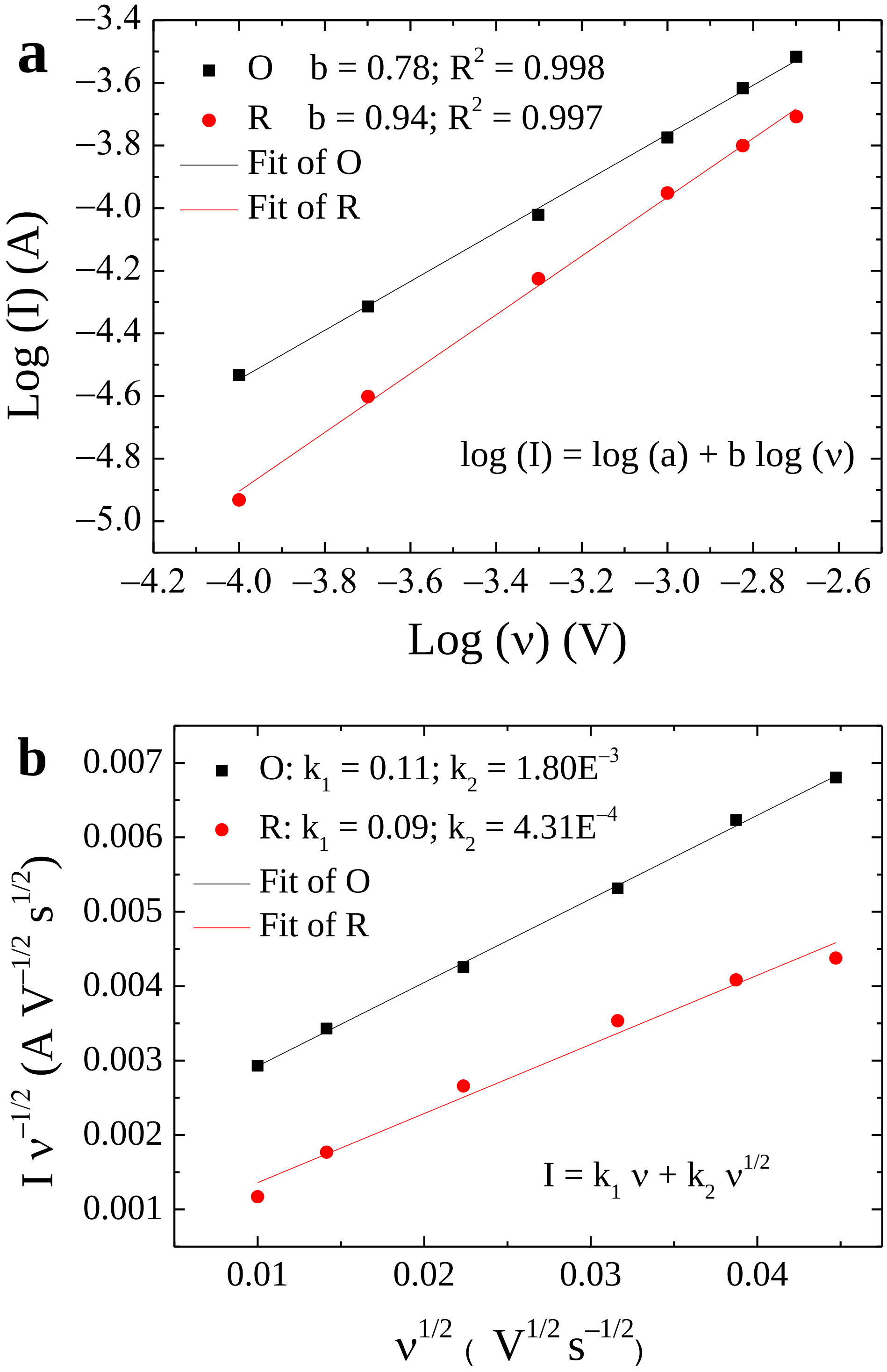

Supplementary Fig. 22 Kinetic analysis of MoS_2@C-PNR electrode. **a** Current response versus scan rate (log I vs. log v) plotted at each redox peak. **b** I $v^{-1/2}$ versus $v^{1/2}$ plotted at different voltages. The data are calculated based on the CV curves with different scan rate in Supplementary Fig. 9.

Supplementary Tables

Supplementary Table 1 A performance comparison of the mostly used electrolytes in MBs. In all cases, Pt was used as the working electrode.

Electrolyte	Overpotential (V)	Oxidation stability (V)	Corrosive	Ref
0.25 M $Mg(AlCl_2BuEt)_2$/THF	0.3	2.2	Yes	[1]
0.4 M PhMgCl-$AlCl_3$/THF	0.25	3.5	Yes	[2]
1.8 M $[Mg_2Cl_3][HMDSAlCl_3]$/DEG	0.5	3.2	Yes	[3]
0.5 M $Mg(TFSI)_2$/DME	>2.0	-	No	[4]
$Mg(TFSI)_2$-$MgCl_2$/DME	0.5	3.2	Yes	[4]
0.4M MgBOR/DME	0.32	4.5	No	[5]

Supplementary Note 1

In order to clarify that carbon contribution to the capacity is negligible, pure MoS_2 nanomaterial (s-MoS_2) was synthesized through the same procedure without addition of dopamine. Meanwhile, the carbon species (s-C) were synthesized by hydrothermally treating the dopamine/Tirs solution at 200 °C for 24 h followed by the same sintering procedure. The s-MoS_2 electrode exhibits similar behaviour and offers comparable capacity (~100 mA h g^{-1}) as the MoS_2@C-PNR electrode during galvanostatic cycling (Supplementary Fig. 6a and b). As for s-C electrode (Supplementary Fig. 6c), it only delivers negligible capacity (<5 mA h g^{-1}) at this condition. The dopamine acts as a regulation agent which helps to obtain MoS_2 nanosheets with smaller sizes. After sintering, the carbon species in MoS_2@C-PNR could improve the electric conductivity of the electrode material.

Supplementary Note 2

In Supplementary Fig. 16, it should be mentioned that the S-K and Mo-L overlap makes the S strongly underestimated. Quantification of Mo is using its K edge. The Mo:Mg:O ≈ 1:2:6, indicating a high capacity in the activated material shell (under ~100 nm depth of the surface). The Mg/Mo ratio is lower than the value determined from XPS (1:4.9), indicating a gradual magnesiation process from surface to bulk. The Mg/O ratio is lower than that in the electrolyte molecule (1:6). It might imply an intercalation of uncomplete $[Mg(DME)_3]^{2+}$. However, there is a strong potential to underestimate the O content due to the low energy of the O-K X-ray makes them easily being absorbed by the sample.

Supplementary Note 3

In the equivalent circuit (Supplementary Fig. 20c), R_1 could be attributed to the electrolyte resistance while R_2 and R_3 are the charge transfer resistance for cathode and anode respectively. The low R_1 value corresponds to a high conductivity of the MgBOR/DME electrolyte which has been discussed in our previous works[5,6] already. For the anode, the high conductivity of the magnesium anode allows for fast electron transfer so that a double layer capacitance (C_3) is paralleling to R_3. This result matches well with the Mg-Mg symmetric cell reported previously.[7] While for the cathode, the capacitor did not behave ideally so that a constant phase element (Q_1) is applied. This is a common phenomenon in real cell impedance measurement. The Warburg impedance (W_2) in cathode results from the diffusion controlled process during de-/intercalation in the bulk part.

Supplementary Note 4

The Mg^{2+} diffusivity was determined by the following equation:

$$D^{GITT} = \frac{4}{\pi\tau} (\frac{m_B V_M}{M_B S})^2 (\frac{\Delta E_S}{\Delta E_\tau})^2$$

As shown in Supplementary Fig. 21a, τ is the constant current pulse time, m_B, V_M, M_B, and S refer to the mass, molar volume, molar mass of the cathode materials, and electrode-electrolyte interface area respectively. ΔE_S is the voltage change in a single-step including the relaxation, while ΔE_τ refers to the difference of cell voltage in a constant current pulse step excluding the IR drop.

Supplementary References

1. Aurbach, D. *et al.* Prototype systems for rechargeable magnesium batteries. *Nature* **407**, 724 (2000).
2. Aurbach, D. *et al.* Progress in Rechargeable Magnesium Battery Technology. *Adv. Mater.* **19**, 4260–4267 (2007).
3. Zhao-Karger, Z. *et al.* Performance Improvement of Magnesium Sulfur Batteries with Modified Non-Nucleophilic Electrolytes. *Adv. Energy Mater.* **5**, 1401155 (2015).
4. Shterenberg, I. *et al.* Evaluation of $(CF_3SO_2)_2N^-$ (TFSI) Based Electrolyte Solutions for Mg Batteries. *J. Electrochem. Soc.* **162**, A7118–A7128 (2015).
5. Zhao-Karger, Z. *et al.* Toward Highly Reversible Magnesium-Sulfur Batteries with Efficient and Practical $Mg[B(hfip)_4]_2$ Electrolyte. *ACS Energy Lett.* **3**, 2005–2013 (2018).
6. Zhao-Karger, Z., Gil Bardaji, M. E., Fuhr, O. & Fichtner, M. A new class of non-corrosive, highly efficient electrolytes for rechargeable magnesium batteries. *J. Mater. Chem. A* **5**, 10815–10820 (2017).
7. Tutusaus, O., Mohtadi, R., Singh, N., Arthur, T. S. & Mizuno, F. Study of Electrochemical Phenomena Observed at the Mg Metal/Electrolyte Interface. *ACS Energy Lett.* **2**, 224–229 (2017).
